# The Bayesian-Based Area under the Curve of Vancomycin by Using a Single Trough Level: An Evaluation of Accuracy and Discordance at Tertiary Care Hospital in KSA

**DOI:** 10.3390/healthcare11030362

**Published:** 2023-01-27

**Authors:** Abdullah M. Alzahrani, Anjum Naeem, Rami M. Alzhrani, Manar A. Harbi, Sarah A. Alghamdi, Shahid Karim, Ahmed S. Ali, Ghusun Alsenaini, Hani Hasan, Ayed A. Alkatheeri, Samah S. Basudan, Yahya A. Alzahrani

**Affiliations:** 1 Pharmaceutical Care Department, Ministry of National Guard—Health Affairs, Jeddah 22384, Saudi Arabia; 2 King Abdullah International Medical Research Center, Jeddah 21423, Saudi Arabia; 3 College of Medicine, King Saud bin Abdulaziz University for Health Sciences, Jeddah 22384, Saudi Arabia; 4 Department of Pharmaceutics and Industrial Pharmacy, College of Pharmacy, Taif University, Taif 21944, Saudi Arabia; 5 Faculty of Pharmacy, King Abdulaziz University, Jeddah 21589, Saudi Arabia; 6 Department of Pharmacology, Faculty of Medicine, King Abdulaziz University, Jeddah 21589, Saudi Arabia; 7 Drug Information Center, Department of Pharmacy, East Jeddah Hospital, Ministry of Health, Jeddah 23816, Saudi Arabia; 8 Department of Pharmacy, King Abdullah Medical Complex, Ministry of Health, Jeddah 23816, Saudi Arabia

**Keywords:** PrecisePK, AUC_0–24_, vancomycin, discordance, trough level, Bayesian software

## Abstract

The AUC_0–24_ is the most accurate way to track the vancomycin level while the C_min_ is not an accurate surrogate. Most hospitals in Saudi Arabia are under-practicing the AUC-guided vancomycin dosing and monitoring. No previous work has been conducted to evaluate such practice in the whole kingdom. The current study objective is to calculate the AUC_0–24_ using the Bayesian dosing software (PrecisePK), identify the probability of patients who receive the optimum dose of vancomycin, and evaluate the accuracy and precision of the Bayesian platform. This retrospective study was conducted at King Abdulaziz medical city, Jeddah. All adult patients treated with vancomycin were included. Pediatric patients, critically ill patients requiring ICU admission, patients with acute renal failure or undergoing dialysis, and febrile neutropenic patients were excluded. The AUC_0–24_ was predicted using the PrecisePK platform based on the Bayesian principle. The two-compartmental model by Rodvold et al. in this platform and patients’ dose data were utilized to calculate the AUC_0–24_ and trough level. Among 342 patients included in the present study, the mean of the estimated vancomycin AUC_0–24_ by the posterior model of PrecisePK was 573 ± 199.6 mg, and the model had a bias of 16.8%, whereas the precision was 2.85 mg/L. The target AUC_0–24_ (400 to 600 mg·h/L) and measured trough (10 to 20 mg/L) were documented in 127 (37.1%) and 185 (54%), respectively. Furthermore, the result demonstrated an increase in odds of AUC_0–24_ > 600 mg·h/L among trough level 15–20 mg/L group (OR = 13.2, *p* < 0.05) as compared with trough level 10–14.9 mg/L group. In conclusion, the discordance in the AUC_0–24_ ratio and measured trough concentration may jeopardize patient safety, and implantation of the Bayesian approach as a workable alternative to the traditional trough method should be considered.

## 1. Introduction

Vancomycin has been used as first-line therapy against *Methicillin-resistant Staphylococcus aureus* (MRSA) infection [1]. The dosing of vancomycin is one of the significant challenges in clinical practice due to the individual pharmacokinetic (PK) variability, the low bactericidal activity due to poor tissue penetration, and the toxicity that arises from high doses [2]. The 2009 vancomycin guideline was based on identifying the ratio of AUC_0–24_ to MIC (AUC_0–24_/MIC) as the most accurate way to track the vancomycin Pharmacokinetic/ Pharmacodynamic [3]. However, the Recent guideline has revealed that C_min_ is not an accurate surrogate as it underestimates the vancomycin level by 25% [4]; consequently, a higher dose of vancomycin is needed to enhance the regimen efficiency. Increasing the vancomycin dose may lead to nephrotoxicity, which is a major risk factor in enhancing the mortality in *S. aureus bacteremia* (SAB) [1,5]. Thus, using AUC_0–24_/MIC-guided monitoring will play a crucial role in providing a rapid and accurate AUC_0–24_/MIC prediction and reducing nephrotoxicity compared to C_min_-guided monitoring [6,7]. Tsutsuura et al. reported that the low treatment failure is associated with high AUC_0–24_ (cut off, 400 mg·h/L ± 15%), while a high risk of nephrotoxicity is associated with AUC_0–24_ (cut off, 600 mg·h/L ± 15%) [7].

The 2020 guidelines recommend targeting a vancomycin ratio of 400 to 600 mg·h/L with an assumption of MIC of the MRSA is 1mg/L [6]. Therefore, there is an unmet need to precisely predict the AUC_0–24_ with minimum blood sampling to improve the vancomycin efficacy. The computerized Bayesian forecasting platform can be utilized to monitor vancomycin dosing. The Bayesian method uses the subject information to integrate the population PK model and specifically estimates the individual PK parameters to calculate the patient AUC_0–24_ [8]. The main pro of using the Bayesian program is precisely estimating the AUC_0–24_ with minimum concentration data; therefore, the flexibility of sample collection and achieving therapeutic target increase while the patient burden and drug toxicity will be minimized [9].

To accurately estimate AUC_0–24_ using the Bayesian method, it prefers to obtain two PK samples; however, relying on large samples to integrate the PK model based on C_min_ can be sufficient to generate an accurate AUC_0–24_ estimate [6,10]. The 2020 consensus guideline states that the two data points might be required for those patients who need a high precision due to hard-to-treat MRSA infection or patients suffering from kidney diseases [6,11]. However, relying on a trough level concentration only can be sufficient to predict AUC_0–24_ using the Bayesian approach if rich samples are used [6].

To our knowledge, no prior studies have been conducted to evaluate such practice in the whole kingdom. The current study objective is to calculate the AUC_0–24_ from single point concentration (trough concentration) using the Bayesian dosing software (PrecisePK), the accuracy and precision of this software in determining the trough level are also estimated. Assessing the prediction and accuracy of the Bayesian software will enable clinical providers to identify the percentage of patients who receive the optimum dose of vancomycin. Moreover, the current study will highlight the association between the attainment of AUC_0–24_ ≥ 600 mg·h/L and trough levels of 10–14.9 mg/L and 15–20 mg/L, respectively. We also sought to identify common factors that increase the probability of the target AUC_0–24_ = 400–600 mg·h/L group compared to abnormal AUC_0–24_ for which targeted therapeutic drug monitoring (TDM) efforts could be implemented.

## 2. Materials and Methods

### 2.1. Place of Study

This study was carried out at King Abdulaziz medical city, Jeddah (KAMC-J), in the inpatient setting from 1 January 2019 to 31 December 2019.

### 2.2. Study Design, Setting, and Patient Population

This is a retrospective single-center cohort study conducted at KAMC-J, an 800-bed tertiary hospital located in Jeddah, KSA. The KAIMRC’s institutional review board (IRB) approved this study with a number (NRJ21J/241/10). All demographic data, including age, weight, height, serum creatinine, type of treatment (empirical or therapeutic), vancomycin dose, frequency, and trough level measured at a steady state (mg/L) were collected retrospectively using a validated, standardized data collection sheet reviewed by three experts in the same field (two ID clinical pharmacists and infectious disease consultant). Patients with documented infection were identified by using the microbiology database for those who underwent nasal swab MRSA PCR testing or blood and respiratory cultures for MRSA or other gram-positive organisms.

### 2.3. Vancomycin Dosing and Monotiring

Based on the current clinical practice at KAMC—Jeddah, the initial vancomycin dose is 15–20 mg/kg every 8–12 h. using the actual body weight and administered over 90 to 120 min. Serum trough is usually done as a routine for all patients with normal kidney function 30 min before the 4th dose. The subsequent doses were adjusted according to the trough level [12].

### 2.4. Pharmacokinetic Parameters Estimation

The AUC_0–24_ was predicted using the PrecisePK platform (Healthware Inc., San Diego, CA, USA) based on the Bayesian principle. The two-compartmental model by Rodvold et al. [13] in this platform and patients’ dose data were utilized to calculate the AUC_0–24_ and trough. The Cockcroft-Gault equation was computed to calculate the creatinine clearance (CrCl).

### 2.5. Criteria of Inclusion and Exclusion

All adult patients treated with vancomycin empirically or therapeutically for documented or suspected infection were included. Additionally, all the study patients were only included if the dose was within the normal range (15–20 mg/kg/dose), otherwise the patients were excluded.

Pediatric patients, critically ill patients requiring ICU admission, patients with acute or chronic renal failure or undergoing dialysis, and febrile neutropenic patients were excluded. 

### 2.6. Endpoints

#### 2.6.1. Primary Endpoints

- To estimate the vancomycin AUC_0–24_ using Bayesian software and determine the probability (%) of patients who achieved the targeted AUC_0–24_ of 400–600 mg·h/L.
- To evaluate the accuracy and precision of the PrecisePK Bayesian platform in determining the trough level.

#### 2.6.2. Secondary Endpoints

- To compare the association between the attainment of AUC_0–24_ ≥ 600 mg·h/L and trough levels of 10–14.9 mg/L, and 15–20 mg/L, respectively.
- To identify factors for achieving target AUC_0–24_.

### 2.7. Statistical Analysis

Data were analyzed using a statistical package for the social sciences (SPSS Inc., Chicago, IL, USA). Kolmogorov-Smirnov and histogram tests were performed to determine if data were normally distributed. Categorical variables were expressed using frequency and percentages, while the mean ± standard deviation was used to present continuous variables. The Pearson’s coefficient was used to determine the correlation between the actual vancomycin trough levels and calculated trough values by Bayesian software. The accuracy and precision of Bayesian software were assessed by calculating mean absolute percentage error (MAPE) and root mean squared error (RMSE), respectively. MAPE was reported as acceptable when ≤20% [14]. The Bayesian software validity was assessed by using the Bland-Altman graph (measuring bias and limits of agreement). The relationships of covariates on the AUC_0–24_ were determined using stepwise multiple regression analysis. Multinomial logistic regression was performed to estimate adj. odds ratios (adj. ORs) and calculate 95% confidence intervals (CIs) for the factors for low and high AUC_0–24_. 

Within each trough group, the probability (%) of patients who achieved the target AUC_0–24_ of 400–600 or >600 mg·h/L was also determined. The χ^2^ test was performed to determine the association between an AUC_0–24_ of >600 mg·h/L and trough values of 10–14.9 mg/L vs. those of 15 mg/L or greater. All reported *p*-values were 2-sided, and a *p*-value of <0.05 was considered statistically significant.

## 3. Results

The data of 342 patients in the period between January 1st and December 31st, 2019, were collected. Patients were equally distributed male (181 patients, 52.9%) and female (161 patients, 47.1%). 71.3% of the patient started vancomycin empirically, and 28.7% were treated based on documented infection. The most frequent indication for vancomycin was an infection of the skin and soft tissues (30%), followed by pneumonia (14.3%) and bacteremia (11.2%). The mean (±SD) patients’ weight, age, and calculated CrCl at baseline were 68.9 kg ± 20, 57 years ± 19, and 85.6 mL/min ± 42.7, respectively. The mean dose of vancomycin was 1857 mg ± 590, with a mean measured vancomycin trough of 13.6 ± 6.9, and the corresponding calculated trough of 13.38 ± 7.3 (Table 1).

### 3.1. Estimation of AUC_0–24_ and Determine the Probability of Patients Who Achieved Optimal AUC_0–24_

**Figure 1:**
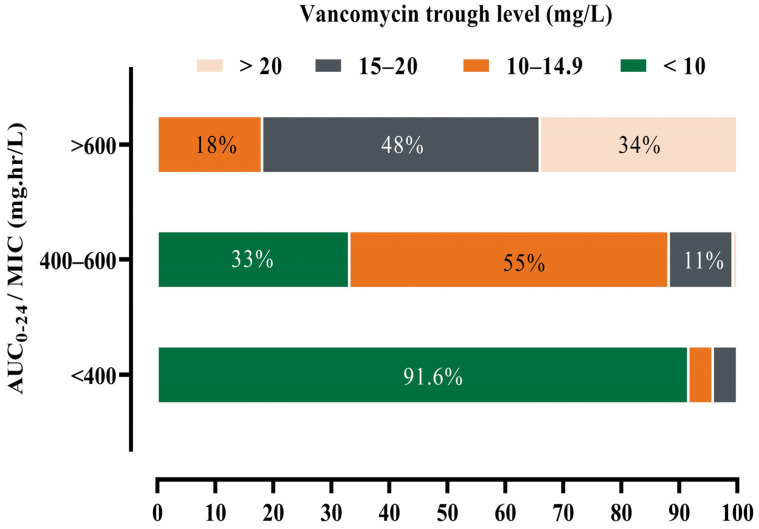
The percentage of patients with vancomycin trough level <10, 10–14.9, 15–20, >20 among each AUC_0–24_ level. low AUC_0–24_ < 400 mg·h/L n = 71 (20.8%), normal AUC_0–24_ 400–600 mg·h/L n = 127 (37.1%) and high AUC_0–24_ > 600 mg·h/L n = 144 (42.1%); AUC: the area under the curve.

The mean of the estimated vancomycin AUC_0–24_ by using the PrecisePK software was 573 ± 199.6 mg·h/L. The target AUC_0–24_ (400 to 600 mg·h/L) and measured trough (10 to 20 mg/L) were documented in 127 (37.1%) and 185 (54%) patients, respectively. Additionally, out of 127 patients, 55% had a trough between 10–14.9 mg/L, while 14 (11%) patients had a trough level of 15–20 mg/L. Moreover, among patients with AUC_0–24_ > 600 mg·h/L, 18 %, and 48% had trough levels 10–14.9 mg/L, 15–20 mg/L, respectively. Figure 1 depicted all computed AUC_0–24_ and measured trough concentration ranges for all the study patients.

### 3.2. Accuracy and Precision of Bayesian Software (Posterior) in Determining the Trough Level

**Figure 2:**
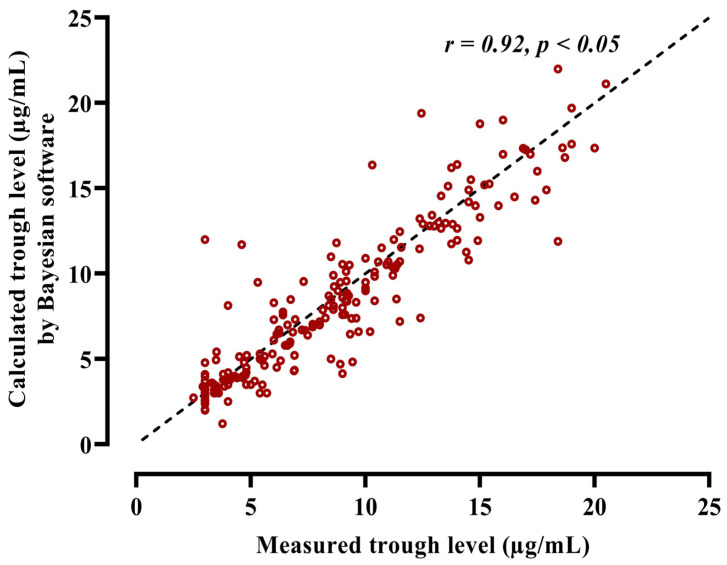
Diagnostic goodness of fit between measured and calculated vancomycin trough levels.

**Figure 3:**
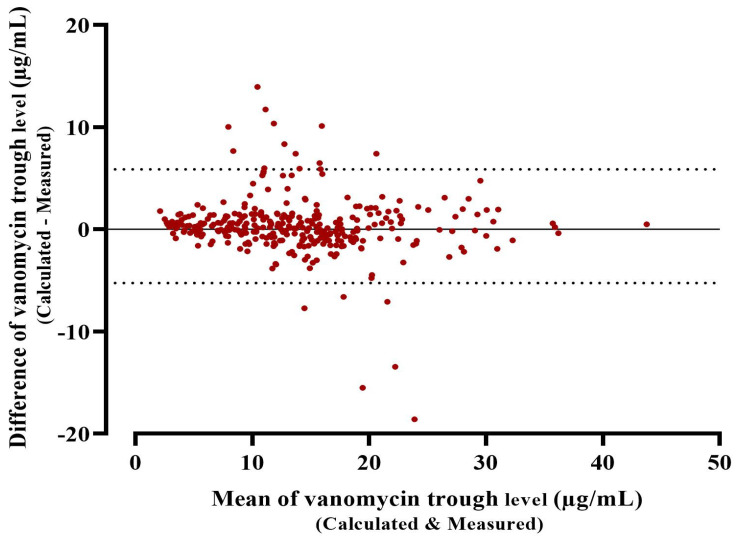
Bland-Altman plot for Bayesian software validity in prediction vancomycin trough. Each sample is represented on the graph by conveying the mean value of the 2 trough levels (*x*-axis) and the difference between the 2 trough levels (*y*-axis). The mean difference was the estimated bias, and the standard deviation (SD) of the differences measured the fluctuations around this mean (outliers being above 1.96 SD diff). Reference lines show the mean difference between time 1 and time 2 (solid line), and dashed lines represent 95% limits of agreement.

The performance of the posterior model of PrecisePK, as evaluated by comparing measured trough level and PrecisePK predicted values, showed a good correlation (r = 0.92) (Figure 2). The PrecisePK platform model by Rodvold et al. had a bias of 16.8%, whereas the precision was 2.85, and the 95% limits of agreement were −5.26 to 5.87 with a mean difference of 0.3 and a bias of 0.3, which indicates that there is no major bias (Figure 3).

### 3.3. Association of AUC_0–24_ ≥600 mg·h/L with Normal Trough Level

A chi-square test of independence was used to assess the association between vancomycin trough level 10–14.9 mg/L and 15–20 mg/L in the attainment of AUC_0–24_ > 600 mg·h/L, and the result showed a significant association between the AUC_0–24_ > 600 mg·h/L and trough level 15–20 mg/L: *p* < 0.05. Furthermore, the result demonstrated an increase in odds of AUC_0–24_ > 600 mg·h/L among trough level 15–20 mg/L group (OR = 13.2, 95% CI = 6.4 to 26.9, *p* < 0.05), as compared with trough level 10–14.9 mg/L group.

### 3.4. Factors for Achieving Target AUC_0–24_

The stepwise regression examined the relationship between AUC_0–24_ and the patient’s demographics to find potential covariates fitting in a linear regression model. Trough level, CrCl, and BMI had significant correlations with AUC_0–24_ (r = 0.9, 0.38, and 0.14 respectively, *p* < 0.05). The following equation summarizes this relation:

AUC_0–24_ = 118.9 + (24.5 × Trough) + (3.2 × TDD) + (3.215 × BMI) − (0.6 × CrCl)

where AUC_0–24_: is area under the plasma concentration-time curve over the last 24-h dosing interval (mg·h/L), trough: is steady-state trough concentration (µg/mL), TDD: is the total daily dose (mg/kg/day), BMI: body mass index (kg/m^2^), CrCl: estimated creatinine clearance using Cockcroft-Gault equation (mL/min).

Furthermore, multivariate logistic regression was used to determine which factors were more likely to classify patients as normal rather than low or high AUC_0–24_. Table 2 provides a summary of the adj. OR and 95% CI of significant factors.

## 4. Discussion

In the current study, the mean of the estimated AUC_0–24_ was 573 ± 199.6 mg·h/L. The AUC_0–24_ was estimated based on single-point concentration (trough level). 

To calculate the AUC_0–24_ with the Bayesian software, it is recommended to obtain two samples (i.e., peak two hours post-infusion and trough 30–60 min before the next dose) [6]. A trough concentration alone may be adequate to predict the AUC_0–24_ using the Bayesian method, but further data from different patient groups are required to prove the validity of employing trough-only measurements. The revised therapeutic drug monitoring guideline recommends that “a trough concentration alone may be sufficient to estimate the AUC_0–24_ with the Bayesian approach” [6,15]. However, Neely et al. reported a satisfactory performance in estimating AUC_0–24_ using only the trough concentration based on a population PK model created utilizing richly sampled concentration data (approx. 6 sample concentrations during a dosing interval) [16]. Similarly, Turner et al. reported that using the trough concentration to estimate AUC_0–24_ will produce an accuracy (range 0.79–1.03) and bias (range 5.1–21.2%) with different Bayesian dose-optimizing software [17]. A recent study by Olney et al. concluded that Bayesian one-concentration approaches may provide an alternate method for predicting AUC_0–24_ and lowering hospital expenditures compared to two-level methods. Moreover, the authors reported a strong correlation between Bayesian two-level and one-level methods (r = 0.93), with an overall 88.5% clinical decision agreement and a low mean difference (MD) between Bayesian and linear AUC_0–24_ methods [18].

In the current study, there is a high degree of variability between a measured trough concentration and the AUC_0–24_ value. Comparing the target AUC_0–24_ dosing method with the trough-based method showed that only 37.1% of the included patients achieved the target AUC_0–24_ 400–600 mg·h/L, while the majority attained AUC_0–24_ >600 mg·h/L. It is interesting to note that the upper limits of the target trough level (15–20 mg/L) in this study were significantly associated with AUC_0–24_ > 600 mg·h/L, as compared to the group of trough level 10–14.9 mg/L. These results are in line with a previous study by Lodie et al. that mentioned that when the relationship between steady-state trough concentration and AUC_0–24_ is examined, a trough concentration will not explain more than 50% of the interindividual variability in AUC_0–24_ (r^2^ = 0.409) [19]. Furthermore, the observed variation between the trough and AUC_0–24_ can be explained in this way. With vancomycin trough values between 15 and 20 mg/L, the likelihood of reaching an AUC_0–24_ of 400 mg·h/L is always 100% without considering the upper range of AUC_0–24_, which varies significantly from patient to patient [19,20]. 

These results provide further support for shifting the practice to the dose and monitoring of vancomycin guided by AUC_0–24_. Two recent studies by Neely et al. and Finch et al. found that the dose and monitoring of vancomycin guided by AUC_0–24_ are associated with less nephrotoxicity compared to trough-based monitoring [9,21]. This discordance in the AUC_0–24_ ratio and measured trough concentration may lead to treatment failure or expose the patient to severe adverse effects. It is highly important to consider the consequences of discordance in clinical decision classification from a safety and efficacy perspective. Two different scenarios may arise; the unmatched classification represents the most important one when the AUC_0–24_ method predicts a supratherapeutic while the trough method gives a reading in the normal range. The problem is that the prescribers will misclassify this case as therapeutic, and there will be no dose adjustment, resulting in nephrotoxicity, and notably that 27.8% of the normal trough cases in our study lie under this scenario. In contrast, the other scenario may affect the clinical improvement when the AUC_0–24_ predicts a subtherapeutic while trough values lying under the therapeutic range. The consequences of such discrepancies may mislead the physician to maintain the dosage although real exposure is inadequate, thus increasing the risk of treatment failure, hospital length of stay, and mortality [7,22,23]. It’s not surprising that the therapeutic effects of the trough and AUC_0–24_ values are different since AUC_0–24_ is the sum of all the times a drug is exposed. However, the trough shows a single point of exposure at the end of the dosing interval. Notably, trough levels are considered a poor detector of AUC_0–24_ ratios, three more recent investigations demonstrated that over 50% with AUC_0–24_ 400–600 mg·h/L had trough values < 15 mg/L [16,24,25].

The trough level, BMI, CrCl, and TDD have been demonstrated to be predictive factors for high AUC_0–24_. Patients with low CrCl levels tend to have high AUC_0–24_ (adj. OR = 0.97). However, high trough level, BMI, and TDD were associated with high AUC_0–24_ (adj. OR = 2.09,1.13, and 1.13 respectively) and vice versa for the low AUC_0–24_ group. No other predictor variables were statistically significant for the development of low and high AUC_0–24_ in this model. No other predictor variables were statistically significant for the development of low and high AUC_0–24_ in this model. These results are in agreement with those of Suzuki et al., who examined predictive factors for high trough concentrations [26].

Overall, the current study has some limitations; first, this study includes the retrospective nature of data collection and reliance on the computerized provider order entry to extract all data points from a single center that limits the ability to generalize the results. Second, the AUC_0–24_ estimation was based on a single point concentration and there was a debate over using a single C_min_ to generate accurate AUC_0–24_ estimates. In addition, the current TDM recommendations at our institution indicate achieving roughly steady-state vancomycin concentrations by the fourth dosage following its initiation. As a result, there is a possibility that certain patients’ trough concentrations were collected before the actual steady state. Finally, the AUC_0–24_: MIC ratio was not evaluated in this study, and we considered MIC = 1 mg/L for all strains.

## 5. Conclusions

The main conclusion that can be drawn is that one-third of the patients with the targeted goal of AUC_0–24_ had a trough level below the targeted goal, and dangerously noted that patients with normal trough levels between 15–20 mg/L had a 13-fold increased risk of AUC_0–24_ > 600 mg·h/L. Despite the limitations abovementioned, the current work might encourage physicians to improve their clinical practice and implantation of the Bayesian approach as a workable alternative to the traditional trough method. Future investigations should be carried toward evaluating the clinical implementation of AUC_0–24_-based dosing using Bayesian software.

## Figures and Tables

**Table 1:** Patient Demographics, Clinical Characteristics, Vancomycin Dose and Monitoring Parameters.

Variables	n (%)
Gender	
Male	181 (52.9)
Vancomycin Indication	
Documented infection	98 (28.7)
Empirical therapy	244 (71.3)
Type of infection	
Pneumonia, unspecified	14 (14.3)
Bacteremia	11 (11.2)
Bone and skin infection	29 (29.6)
	**Mean ± SD**
Age (years)	57 ± 19.2
BMI (Kg/m^2^)	26.6 ± 7.6
Weight (kg)	68.9 ± 20
CrCl (mL/min)	85.6 ± 42.7
TDD (mg/day)	1857 ± 590
Estimated AUC_0–24_ (mg. hr/L) *	573 ± 199.6
Trough level mg/L mean ± SD	13.69 ± 6.9
Calculated trough mg/L *	13.38 ± 7.29

* Calculated by PrecisePK using patient dose data; AUC; area under the curve, BMI: body mass index, CrCl: creatinine clearance, TDD: total daily dose.

**Table 2:** Multinomial logistic regression analysis of the factors for the patient being in the low or high AUC_0–24_ group as compared with normal AUC_0–24_.

Independent Factors	Low AUC_0–24_ vs. Normal AUC_0–24_				High AUC_0–24_ vs. Normal AUC_0–24_			
	β	Adj. OR	(95% CI)	*p*-Value	β	Adj. OR	(95% CI)	*p*-Value
**Trough level**	−0.591	0.554	0.463–0.662	<0.05	0.741	2.09	1.724–2.551	<0.05
**CrCl**	0.015	1.015	1.001–1.030	<0.05	−0.025	0.976	0.959–0.992	<0.05
**BMI**	−0.144	0.866	0.79–0.949	<0.05	0.119	1.126	1.057–1.2	<0.05
**TDD**	−0.014	0.866	0.802–0.936	<0.05	0.122	1.13	1.052–1.213	<0.05

AUC; area under the curve, Adj. OR: adjusted odd ratio, 95% CI: confidence interval, BMI: body mass index, CrCl: creatinine clearance, TDD: total daily dose, β: logistic regression coefficient.

## Data Availability

The datasets used and/or analyzed during the present study are available from the corresponding author on reasonable request.

## References

[1] Hassoun A., Linden P.K., Friedman B. (2017). Incidence, Prevalence, and Management of MRSA Bacteremia across Patient Populations—A Review of Recent Developments in MRSA Management and Treatment. Crit. Care.

[2] Rybak M.J. (2006). The Pharmacokinetic and Pharmacodynamic Properties of Vancomycin. Clin. Infect. Dis..

[3] Rybak M., Lomaestro B., Rotschafer J.C., Moellering R., Craig W., Billeter M., Dalovisio J.R., Levine D.P., Reilly C. (2009). Therapeutic Monitoring of Vancomycin in Adult Patients: A Consensus Review of the American Society of Health-System Pharmacists, the Infectious Diseases Society of America, and the Society of Infectious Diseases Pharmacists. Am. J. Health-Syst. Pharm..

[4] Holubar M., Meng L., Deresinski S. (2016). Bacteremia Due to Methicillin-Resistant Staphylococcus Aureus: New Therapeutic Approaches. Infect. Dis. Clin. N. Am..

[5] Jacob J.T., DiazGranados C.A. (2013). High Vancomycin Minimum Inhibitory Concentration and Clinical Outcomes in Adults with Methicillin-Resistant Staphylococcus Aureus Infections: A Meta-Analysis. Int. J. Infect. Dis..

[6] Rybak M.J., Le J., Lodise T.P., Levine D.P., Bradley J.S., Liu C., Mueller B.A., Pai M.P., Wong-Beringer A., Rotschafer J.C. (2020). Therapeutic Monitoring of Vancomycin for Serious Methicillin-Resistant Staphylococcus Aureus Infections: A Revised Consensus Guideline and Review by the American Society of Health-System Pharmacists, the Infectious Diseases Society of America, the Pediatr. Clin. Infect. Dis..

[7] Tsutsuura M., Moriyama H., Kojima N., Mizukami Y., Tashiro S., Osa S., Enoki Y., Taguchi K., Oda K., Fujii S. (2021). The Monitoring of Vancomycin: A Systematic Review and Meta-Analyses of Area under the Concentration-Time Curve-Guided Dosing and Trough-Guided Dosing. BMC Infect. Dis..

[8] Donagher J., Martin J.H., Barras M.A. (2017). Individualised Medicine: Why We Need Bayesian Dosing. Intern. Med. J..

[9] Neely M.N., Kato L., Youn G., Kraler L., Bayard D., Van Guilder M., Schumitzky A., Yamada W., Jones B., Minejima E. (2018). Prospective Trial on the Use of Trough Concentration versus Area under the Curve to Determine Therapeutic Vancomycin Dosing. Antimicrob. Agents Chemother..

[10] Phillips C.J. (2014). Questioning the Accuracy of Trough Concentrations as Surrogates for Area under the Curve in Determining Vancomycin Safety. Ther. Adv. Drug Saf..

[11] Ueda T., Takesue Y., Nakajima K., Ichiki K., Ishikawa K., Yamada K., Tsuchida T., Otani N., Takahashi Y., Ishihara M. (2022). Validation of Vancomycin Area under the Concentration—Time Curve Estimation by the Bayesian Approach Using One-Point Samples for Predicting Clinical Outcomes in Patients with Methicillin-Resistant Staphylococcus Aureus Infections. Antibiotics.

[12] Alzahrani A.M., Naeem A., Alwadie A.F., Albogami K., Alzhrani R.M., Basudan S.S., Alzahrani Y.A. (2021). Causes of Vancomycin Dosing Error; Problem Detection and Practical Solutions; A Retrospective, Single-Center, Cross-Sectional Study. Saudi Pharm. J..

[13] Rodvold K.A., Blum R.A., Fischer J.H., Zokufa H.Z., Rotschafer J.C., Crossley K.B., Riff L.J. (1988). Vancomycin Pharmacokinetics in Patients with Various Degrees of Renal Function. Antimicrob. Agents Chemother..

[14] Sheiner L.B., Beal S.L. (1981). Some Suggestions for Measuring Predictive Performance. J. Pharmacokinet. Biopharm..

[15] Oda K., Hashiguchi Y., Kimura T., Tsuji Y., Shoji K., Takahashi Y., Matsumoto K., Kawamura H., Saito H., Takesue Y. (2021). Performance of Area under the Concentration-Time Curve Estimations of Vancomycin with Limited Sampling by a Newly Developed Web Application. Pharm. Res..

[16] Neely M.N., Youn G., Jones B., Jelliffe R.W., Drusano G.L., Rodvold K.A., Lodise T.P. (2014). Are Vancomycin Trough Concentrations Adequate for Optimal Dosing?. Antimicrob. Agents Chemother..

[17] Turner R.B., Kojiro K., Shephard E.A., Won R., Chang E., Chan D., Elbarbry F. (2018). Review and Validation of Bayesian Dose-Optimizing Software and Equations for Calculation of the Vancomycin Area under the Curve in Critically Ill Patients. Pharmacotherapy.

[18] Olney K.B., Wallace K.L., Mynatt R.P., Burgess D.S., Grieves K., Willett A., Mani J., Flannery A.H. (2022). Comparison of Bayesian-Derived and First-Order Analytic Equations for Calculation of Vancomycin Area under the Curve. Pharmacotherapy.

[19] Pai M.P., Neely M., Rodvold K.A., Lodise T.P. (2014). Innovative Approaches to Optimizing the Delivery of Vancomycin in Individual Patients. Adv. Drug Deliv. Rev..

[20] Patel N., Pai M.P., Rodvold K.A., Lomaestro B., Drusano G.L., Lodise T.P. (2011). Vancomycin: We Can’t Get There from Here. Clin. Infect. Dis..

[21] Finch N., Zasowski E., Murray K., Mynatt R., Zhao J., Yost R., Pogue J., Rybak M. (2017). A Quasi-Experiment to Study the Impact of Vancomycin Area under the Concentration-Time Curve-Guided Dosing on Vancomycin-Associated Nephrotoxicity. Antimicrob. Agents Chemother..

[22] Song K.-H., Kim H.B., Kim H., Lee M.J., Jung Y., Kim G., Hwang J.-H., Kim N.-H., Kim M., Kim C.-J. (2015). Impact of Area under the Concentration-Time Curve to Minimum Inhibitory Concentration Ratio on Vancomycin Treatment Outcomes in Methicillin-Resistant Staphylococcus Aureus Bacteraemia. Int. J. Antimicrob. Agents.

[23] Lee B.V., Fong G., Bolaris M., Neely M., Minejima E., Kang A., Lee G., Gong C.L. (2021). Cost-Benefit Analysis Comparing Trough, Two-Level AUC and Bayesian AUC Dosing for Vancomycin. Clin. Microbiol. Infect..

[24] Ghosh N., Chavada R., Maley M., van Hal S.J. (2014). Impact of Source of Infection and Vancomycin AUC0-24/MICBMD Targets on Treatment Failure in Patients with Methicillin-Resistant Staphylococcus Aureus Bacteraemia. Clin. Microbiol. Infect..

[25] Hale C.M., Seabury R.W., Steele J.M., Darko W., Miller C.D. (2017). Are Vancomycin Trough Concentrations of 15 to 20 Mg/L Associated with Increased Attainment of an AUC/MIC ≥ 400 in Patients with Presumed MRSA Infection?. J. Pharm. Pract..

[26] Suzuki A., Hamada Y., Ikeda H., Tanaka H., Yanagihara M., Namiki M., Watanabe T., Sasaki T. (2021). Comparison of Trough Concentration and Area under the Curve of Vancomycin Associated with the Incidence of Nephrotoxicity and Predictors of a High Trough Level. J. Infect. Chemother..

